# Children’s Body Mass Index Depending on Dietary Patterns, the Use of Technological Devices, the Internet and Sleep on BMI in Children

**DOI:** 10.3390/ijerph17207492

**Published:** 2020-10-15

**Authors:** Anna Bartosiewicz, Edyta Łuszczki, Maciej Kuchciak, Gabriel Bobula, Łukasz Oleksy, Artur Stolarczyk, Katarzyna Dereń

**Affiliations:** 1 Institute of Health Sciences, Medical College of Rzeszow University, 35-959 Rzeszow, Poland; abartosiewicz@ur.edu.pl (A.B.); kderen@ur.edu.pl (K.D.); 2 Institute of Physical Culture Sciences, Medical College of Rzeszow University, 35-959 Rzeszow, Poland; mkuchciak@ur.edu.pl (M.K.); gbobula@ur.edu.pl (G.B.); 3 Orthopaedic and Rehabilitation Department, Medical University of Warsaw, 02-091 Warszawa, Poland; loleksy@oleksy-fizjoterapia.pl (Ł.O.); artur.stolarczyk@wum.edu.pl (A.S.); 4 R&D, Oleksy Medical & Sports Sciences, 37-100 Łańcut, Poland

**Keywords:** body composition, children, dietary patterns, obesity, sleep health

## Abstract

Due to the increase in overweight as well as obesity in children, the researchers undertook the studies to determine the occurrence of these irregularities and identify the factors leading to them. The study aimed to assess the body mass index of the children subcategorized and compared depending on the dietary patterns, the use of technical devices, the Internet, and sleeping habits. The study group consisted of 376 children (189 girls and 187 boys) aged 6 to 15. The body composition estimates were obtained with the use of a foot-to-foot bioelectrical impedance analysis, the body height was measured by means of a stadiometer, and blood pressure was monitored. The research questionnaire was distributed among the surveyed and included the question concerning the children’s lifestyle, eating and sleeping habits, the use of electronic devices, the Internet, and socio-demographic data. As indicated, the number of sleeping hours per day significantly negatively correlated with body mass index (BMI), whereas the frequency of using the smartphone had a positive correlation with BMI. The children who sleep less and spend more time using the smartphone had higher BMI values. Bearing in mind the conditions that may have an influence on the BMI of early-school age children, the emphasis must be placed on healthy lifestyle education among children and parents alike.

## 1. Introduction

Due to the increase in children’s overweight and obesity, the researchers undertook the study to determine the occurrence of these irregularities and identify the factors leading to them [1,2]. Overweight and obesity are serious problems in a number of countries and have a considerable effect on both children and adolescents. The diet-related diseases and emotional problems include low self-esteem, lack of self-acceptance, high level of anxiety and aggression, and, in extreme cases, depression. They can be disruptive to social life, especially in a peer group, and in the case of children, obesity can be a factor leading to social isolation [3,4,5,6]. 

According to the experts from the International Obesity Task Force (IOTF), over 150 million schoolchildren have an increased body mass, of whom 45 million are aged 5 to 17. In addition, there is an increase of about 450,000 overweight children and about 100,000 children with obesity every year [7,8]. In Poland, every fifth boy and every seventh school-age girl suffer from overweight and obesity [9,10]. Long-term research in this field was conducted in our region (Podkarpackie Voivoideship, southeast Poland) by Mazur et al., which showed a significant increase in the occurrence of overweight and obesity in children as well as gender differences in a given age group. There was a decreasing obesity incidence in girls and a statistically significant increase in the incidence of overweight in boys [11]. Yet in other research concerning the matter in question, Knai et al. reported a high level of overweight and obesity with an increasing tendency among the population of adults and children, not only in Western but also in Eastern Europe [12]. Although the research indicates that obesity in early childhood does not always lead to problems with excessive body mass in adulthood, it is a leading factor, and the risk of obesity increases more than threefold if a child or a teenager is obese [13,14,15]. The progress of civilization as well as technology and the limitation of physical activity in everyday life resulting from it, when combined with access to highly processed food, are the main factors of the imbalance between the energy intake and consumption, resulting in an increased body mass [16]. The dietary patterns were defined by the 2015 Dietary Guidelines Advisory Committee (U.S. Department of Agriculture, Agricultural Research Service) as *the quantities, proportions, variety or combination of different foods, drinks, and nutrients (when available) in diets and the frequency with which they are consumed* [17]. Healthy dietary patterns, in turn, were defined as the diets that are high in vegetables, fruits, whole grains, low and non-fat dairy, lean protein. Furthermore, they are low in saturated fat, trans fat, sodium, and added sugars [17]. To comprehend the relationship between the dietary patterns of a population of children and overweight/obesity, it is important to develop successful strategies that aim at preventing obesity at the population level. Several approaches have been adopted to characterize dietary patterns. The most prospective cohorts to assess diet is one of many standardized self-reported dietary assessment approaches, such as the food frequency questionnaires, 24-h dietary recalls, or multiple-day food records [18].

The children who have to choose between outdoor activities or computer games and communicating using social media platforms are more likely to choose the latter [19,20]. In the past, Dietz and Gortmarker were among the first to show a relationship between watching TV and childhood obesity [20]. According to the researchers, limiting the time spent in front of the TV can significantly prevent the occurrence of overweight and obesity among children [20,21]. While watching TV is less popular nowadays, the time spent in front of a computer monitor and other technological communications devices has increased significantly [21]. Spending many hours online is a factor leading to unconscious snacking and sleep limitation [22,23]. In addition, the research indicates that too short a sleep period and deterioration of sleep quality resulting from emotional participation in various types of games and online meetings, significantly reduce sensitivity, which leads to reduced glucose tolerance [24]. The studies suggest that sleep reduction may stimulate obesity due to the changes in appetite-regulating hormones (decrease in leptin and increase in ghrelin) and cause a higher intake of high-carbohydrate and high-fat products [25]. The lack of sleep may be the cause of the decrease in body immunity, which not only affects the general weakness of the child’s body, lack of concentration, and irritability but also contributes to the development of cardiovascular diseases because of a positive energy balance resulting from the increased desire to eat. The studies show that sleep that is too short (fewer than 5.5 h per day) significantly increases the risk of metabolic syndrome [25,26]. The justification for the research undertaken is more and more noticeable growth in overweight and obesity in children. The measurements carried out among children by the researchers are an excellent opportunity for early detection of irregularities in the composition of the body mass of the subjects. Similarly, the detection of abnormal behavior associated with the lack of physical activity, too much time spent in front of the computer, smartphone, or TV screen, as well as sleeping disorders can be achieved. This provides the chance to meet and educate parents on the correct patterns for their children and protect them from the occurrence of numerous diseases in the future [27,28].

Given the paucity of population-level data concerning the use of new media (tablets or smartphones) and sleep problems in children, the study aimed to assess the body mass index (BMI) of children subcategorized and compared depending on dietary patterns, the use of technical devices, the Internet, and sleeping habits.

## 2. Materials and Methods

### 2.1. Subjects

A cross-sectional study was adopted for the purpose of this work. The study was conducted during the 2019–2020 school year and covered 3 primary schools located in Leżajsk in Podkarpackie Voivoideship (southeast Poland). The town selected from all the towns in the Podkarpackie Voivoideship to participate in the study (southeast Poland) was chosen at random via an algorithm. The study was conducted from 10 February 2020 until 2 March 2020. After obtaining the consent of the mayor of the town and school directors, all primary schools in the town were included in the study. These schools represent a similar profile of students (public schools). Then a meeting was held with the parents of the children, and consent for each child’s participation in the study was obtained. Invitations to participate in the study were given to 431 parents or guardians of children attending the school selected. The inclusion criteria for children were to be aged from 6 to 15, the attendance of one of the schools, and parents’ acceptance to their children to participate in the study (i.e., all children attending primary school). Currently, there is an 8-year education system in primary school, attended by children aged 6 to 15. We divided our study group into 2 subgroups: children and adolescents, to reflect differences in dietary patterns, sleep, and usage of media in those groups. The study criteria excluded children who suffered from chronic diseases affecting the body mass (e.g., Prader–Willi syndrome, Cushing’s Syndrome/Disease, Down syndrome), and implanted pacemaker (contraindications for bioimpedance testing). The children participating in the study were a representative sample of the population of southeast Poland. The consent of 395 parents was obtained for participation in this study. Of those, 18 were excluded from the study for the following reasons: the functional state that prevents someone from self-maintenance in a standing position (n = 3), the failure to return or complete the survey (n = 4), and refusal to participate in the study (n = 11). The study participants and their legal guardians received verbal and written information about the objectives, nature, he possible risks and benefits of the study. The study was conducted after obtaining written permission from the children’s parents and the children themselves. Finally, the study group consisted of 376 pupils (189 girls and 187 boys) aged 6 to 15.

The body height was measured three times to the nearest 0.1 cm using a portable stadiometer Seca 213. The measurements were performed under standard conditions, barefoot in an upright position. The average figure of the three measurements was used in the analyses. The body mass was assessed with an accuracy of 0.1 kg using a body composition analyzer (BC-420, Tanita). The body mass index (BMI) was calculated as weight (kg)/height (m2). Based on the BMI values, the BMI percentile of the individual participants was calculated. The BMI z-score was also calculated. The World Health Organization (WHO) child growth standard was adopted based on the 2007 WHO Reference, which states that children aged 6 to 19 are overweight and obese with the excess weight of over 1 SD and 2 SD, respectively, and underweight when under 2 SD [29].

### 2.2. Bioelectrical Impedance

The body composition estimates were obtained using the Tanita device (BC-420). All measurements were performed with a frequency of 50 kHz and in the early morning, according to the manufacturer’s guidelines. The measurements were taken after an overnight fasting period (for at least 8 h). The participants stood on the platform barefoot, upright, on straight legs, and after having ascertained that the front of the feet touched the front electrodes and rear parts, the rear electrodes. The body fat (%), fat-free mass (kg), and total body water (kg) were analyzed.

### 2.3. Arterial Blood Pressure

The blood pressure was measured by trained staff three times according to the recommendations of the National High Blood Pressure Education Program Working Group in Children and Adolescents (NHBPEP) [30], by means of the Welch Allyn 4200B-E2 blood pressure meter (Aston Abbotts, UK). The measurements were performed in a sitting position, with the child’s right arm bent at 90 degrees at the heart level and supported on a fixed surface, in a calm and isolated room in the selected schools. During preparation, the child remained at rest for at least 5 min, and the child was instructed not to talk during the measurement. The cuff-size was appropriate for children’s range of arm-circumferences. The average of three measurements was calculated for each person tested. The systolic and diastolic pressure values were used for the analysis.

### 2.4. Questionnaires

The research questionnaire prepared by the authors for the purpose of this study was used. After obtaining the consent of the mayor of the town and the directors of all the primary schools for conducting the research, the information for the pupils and their parents or guardians was sent via the electronic journal. The message included the data on the planned research and a model document for the consent to participate in the study. Participation in the study was voluntary and anonymous. The results were coded as follows: the number of the school the student attended, the number of the class, and the individual number of the student were known only to the student and his parents. The parents who expressed their consent for their children to participate were asked to complete an online questionnaire with their children (aged 6–12). The older children (aged 13–15) were asked to complete the online questionnaire on their own. Each parent or/and a child received a personal survey link. The results were automatically saved on a hard disk. The questionnaire was checked at the survey center for completeness, and missing questions were added by conducting an interview. The questionnaire used in the study covered the information referring to the children’s lifestyle, eating and sleeping habits, the use of technical devices and the Internet, and socio-demographic data. The data were collected using a survey consisting of 4 sections. The survey was designed to take approx. 25 min to complete.

The questionnaire first concerned demographic data (the child’s age, gender). Next, the questions were related to the child’s sleeping behavior: the amount of time spent sleeping at night and during the day and sleep quality. The following questions were asked in this section: How many hours do you sleep at night within one school week? How many hours do you sleep during a 24-h period over the weekend? Over the past month, how would you rate your sleep quality in general? (Answers included: very good, fairly good, fairly bad, very bad). The third part of the questionnaire dealt with the use of technical devices and the Internet by children: How many hours do you usually spend watching movies or programs on the Internet (on an iPad, tablet, computer, smartphone) or TV per day during weekdays? How many hours do you usually spend watching watch movies or programs on the Internet (on an iPad, tablet, computer, smartphone) or on TV during weekends? How many hours do you usually spend playing games (on the computer/ game console/ smartphone/ iPad, etc.) per day during weekdays? How many hours do you usually spend playing games (on the computer/ game console/ smartphone/ iPad, etc.) during the weekend? Possible answers included: not at all, less than 30 min. per day, 30 min–2 h per day, about 2–3 h per day, about 3–6 h per day, more than 6 h per day. The last question checking the use of technological devices and the Internet was, how often do you use a smartphone on a regular day? (I don’t have access to a smartphone, fewer than 5 times a day, 6-10 times a day, 11–20 times a day, 21–50 times a day, 51–100 times a day, more than 100 times per day). Physical activity was examined with the question: Over the last week, how many days have you performed 60 min or more of physical activity that was enough to increase your breathing rate? This may include doing sport, exercising, brisk walking, or cycling for recreational purposes or on the way to these places. The possible answers were 0 to 7 days.

### 2.5. Food Frequency Questionnaire (FFQ)

The Food Frequency Questionnaire (FFQ) was completed by the participants as a part of the survey. The food items considered in the FFQ were the major and commonly consumed food items. The FFQ was presented with a 7-point scale, e.g., How many times did you eat or drink the following food items last month? The possible answers were: never/less than once a week, 1–3 times a week, 4–6 times a week, once a day, twice a day, 3 times a day, 4 or more times a day. The individuals were asked to report the frequency of food and drink consumption over the last month.

The results of the test and instructions on how to interpret the given results were distributed to the parents in sealed envelopes, signed in accordance with the specially generated code, i.e., school number, class number, and individual pupil number (the questionnaire template is attached as Supplementary Materials).

Cronbach’s α of 0.917 indicated high reliability. It showed that the questions concerning the consumption of the selected products accurately assessed the nutritional behavior of the respondents. Cronbach’s alpha for the frequency of using a computer, smartphone, and playing computer games using a stationary or portable device was calculated separately and amounted to 0.766. The reliability of the questions on the sleep quality and sleep period was calculated separately. Cronbach’s alpha of 0.505 was obtained. This value was below the postulated value of 0.7, yet at a satisfactory level, and the questions aiming at sleep assessment positively correlated with each other (in the range of 0.107 and 0.393). The overall power of the test was 95%, as the possible margin of error was 5%. For the calculation of the study power, the number of children in Poland aged 6 to 15 was assumed to be 4 million according to the Central Statistical Office (Statistics Poland, Demographic Department) in 2019 [31], the reliability of the developed tools was satisfactory, and the research power was high.

### 2.6. Statistical Analysis

The results of the study were obtained using the descriptive statistics: number (*n*), %, (mean), Me—median, and standard deviation (SD). The Student’s *t*-test was used for independent variables; alternatively, non-parametric Mann–Whitney U test was used. Additionally, a linear regression analysis was used to model a continuous variable–frequency of BMI. Using the stepwise forward regression procedure, the selection of factors in a statistically significant way describing the level of BMI was made. The presented results were obtained using the principal component analysis (PCA) method of data reduction, which allowed the presentation of original observable variables using the linear component combinations. The PCA was calculated by means of the main component method, with Oblim rotation, and the components were achieved by the Anderson–Rubin method. The statistical significance was established as a *p*-value of less than 0.05. The calculations were performed with the Statistica 13.1 (StatSoft, Inc., Tulsa, OK, USA).

### 2.7. Ethics

The study was approved by the institutional Bioethics Committee at the University of Rzeszow from 2.12.2015 (Resolution No. 15/12/2015) and all appropriate administrative bodies.

## 3. Results

### 3.1. Characteristics of the Study Group

The study tested 376 primary school students, including 187 boys (49.7%) and 189 girls (50.3%). It was found that among children aged 6 to 10, the fat tissue (%) was significantly higher in girls (22.0) than in boys (19.6). In the 11–15 age group, it was noticed that the fat tissue (%) was also higher in girls (23.8 vs. 15.9), while boys had higher fat-free mass values than girls (42.1 vs. 37.6), and the total body water (30.8 vs. 27.5). The descriptive characteristics of the study sample are shown below (Table 1).

The majority of all the respondents were people with normal body mass (279 people equals 74.2%). In the study group, 13 pupils (3.5%) were underweight, 72 subjects (19.1%) were overweight, and 12 pupils (3.2%) were obese (Table 2).

### 3.2. Findings

The relationship between the BMI and the selected variables is presented in Table 3 and Table 4. The study demonstrated that the BMI correlated significantly with the systolic and diastolic pressure in children aged 6 to 10. The overweight or/and obese children had higher systolic and diastolic pressure. In this age group, a statistically significant difference was also shown for the time of using a smartphone on a regular day. The children who used a smartphone more often had a higher BMI value. In children aged 11 to 15, a statistically significant difference was found for the systolic pressure. The children with overweight/obesity have higher systolic pressure (Table 3 and Table 4).

The distribution of the BMI (kg/m^2^) variable was normal. Therefore, the ln (x) transformation was used. Hence, the parametric methods (linear stepwise regression) were used. The following parameters were standardized using the factor analysis: the use of electronic devices and the Internet, sleep, and physical activity. The *media* variable (the use of electronic devices and the Internet) was created out of five detailed variables: How many hours do you usually spend watching movies or programs on the Internet (using an iPad, tablet, computer, smartphone) or TV per day during the weekdays? How many hours do you usually spend watching movies or programs on the Internet (using an iPad, tablet, computer, smartphone) or on TV during the weekend? How many hours do you usually spend playing games (on the computer/ game console/ smartphone/ iPad, etc.) per day during the weekdays? How many hours do you usually spend playing games (on the computer/ game console/smartphone/ iPad, etc.) during the weekend? How often do you use a smartphone on a regular day?

Using the factor analysis, two variables were separated from these five variables using the principal components method, which accounted for a total of 75.28% of the variance (the Cronbach’s alpha analysis confirmed the consistency of these five questions, as Cronbach’s alpha = 0.766). The first variable identified in the analysis consisted of three questions (How many hours do you watch movies online during the weekdays and the weekend, and how often do you use your smartphone?). The second component consisted of two questions (How many hours do you spend playing games online during the weekdays and the weekend). Using the Anderson–Rubin method, these two variables were recorded and named accordingly: media—watching movies and using a smartphone, media—playing games.

The sleep variable was created out of three detailed variables, that is, How many hours do you sleep at night within one school week? How many hours do you sleep during a 24-h period over the weekend? Over the past month, how would you rate your sleep quality in general? These three variables accounted for 52.25% of the variance in total, and their consistency was satisfactory (Cronbach’s alpha 0.505). The variable was described as *sleep*.

The fourth variable concerned physical activity, namely the question over the last week, How many days did you perform 60 min or more of physical activity that was enough to increase your breathing rate?

By means of the stepwise linear regression (the criterion of the stepwise methods was the value of F (introduction 3.84; deletion 2.71)), four variables influencing the BMI level were tested.

It was shown that in the majority of children in question, the value of BMI was influenced by sleep and media—playing games online. The children who slept a short period or had worse quality of sleep had higher BMI values (β = −0.18). Similarly, the children who played online games longer at night had a higher body mass index (β = 0.13).

In the group of girls, the BMI value depended solely on the use of electronic devices and the Internet: the longer the girls played online games (β = 0.25) or the longer they watched movies online or used a smartphone (β = 0.15), the higher the BMI value was. In the group of boys, only the sleep variable influenced the BMI level. The boys who slept a short period or had a worse quality of sleep (β = −0.21) had higher BMI values.

In the age group of 6-10, the BMI was influenced by *media—watching* movies online and using a smartphone. The longer the 6–10-year-olds used the electronic devices and the Internet in this way, the higher their BMI was (β = 0.15). In the children aged 11 to 15, the BMI was related to the quality or quantity of sleep (β = −0.17). The lower the sleep quality, or the shorter the sleep, the higher the BMI (Table 5).

In Table 6, the relationship between the body mass category and frequency of food consumption is shown. It was found that the frequency of product consumption did not affect the BMI value significantly. As for the parameters, the same method was chosen, the use of electronic devices and the Internet, sleep, and physical activity. The groups of products were distinguished, then, using the method of the main components, the indicators were created out of these separate groups linked to the consumption of the selected products. The following groups were created, vegetables, drinks (separately fruit juices, carbonated drinks and tea, and separately water), milk and dairy products and eggs, meat and meat products, fish, fats, grain products (two groups), fast food, and sweets. Whether these product groups had an effect on the BMI (expressed as ln (BMI) to maintain the normal distribution of the variable to the linear regression model) was examined. It was demonstrated that the frequency of food intake did not significantly affect the BMI (all models were statistically insignificant (F = 1.45; *p* = 0.149). Thus, it was confirmed by the linear regression that there was no effect of nutrition on the BMI value (Table 6).

## 4. Discussion

There are numerous studies dealing with overweight and obesity in school-age children, yet, few reports compare the relationship between BMI, sleep, and electronic media usage among a population of healthy children. The matter in question is valuable as children’s dietary patterns, technological devices, and Internet usage, and sleeping habits seem to be a key factor in weight reduction and a healthy lifestyle. The epidemic of obesity is currently one of the most serious problems of modern medicine as well as developed countries.

According to the World Health Organization (WHO), there are over 110 million children in the world with excessive body mass. Overweight and obesity were found in 22.3% of children in our study. Kowal et al. [32] examined children aged 3–18 from the city of Krakow in 1983, 2000, and 2010 and observed that the increase in the incidence of overweight and obesity concerned mainly boys while in girls, it remained at a similar level. Meigenetal [33] observed a significant increase in boys’ obesity compared to girls in 1999 and 2006 in Germany. Oblacińska et al. [34] found overweight in 8.1–8.5% and obesity in 2.9–3.6% of boys aged 13–15. Among girls, they were 8.1–10.1% and 5.2–6.2%, respectively. In 2008/2009, Bac et al. [35] conducted an analysis of the nutritional status among 1499 students aged 6–13. The researchers pointed out that the percentage of children with obesity increased in boys from 1.04% in 1971 to 7% in 2009, while in girls, it increased from 0.20% to 3.6%, respectively.

The proper dietary patterns (a diet high in fruits, vegetables, whole grains, low and non-fat dairy, and lean protein) are the basis of a healthy lifestyle. It is important to provide vitamins, minerals, fiber, as well as many important substances, such as plant sterols, flavonoids, and antioxidants, on a regular basis. Their daily intake helps to prevent non-infectious diseases, such as cardiovascular disease, diabetes, and cancer. Insufficient fruit and vegetable intake is a precursor to overweight or obesity [36], while a high fruit and vegetable intake have beneficial effects on preventing excessive weight gain in adulthood [37,38]. There have been few studies that have examined the dietary intake across countries and the BMI in children. A previous study of 34 countries (primarily European ones) participating in the 2001/2002 Health Behavior in School-Aged Children Study reported that the overweight status was not associated with the intake of fruits or vegetables [39]. In our study, no significant correlation between the children’s BMI and the frequency of food intake, including vegetable and fruit consumption, was observed. In addition, other food product consumption did not show any significant correlation between the frequency of their consumption and children’s BMI. Similarly, in the case of physical activity, there was no correlation between the body mass category and physical activity. Salbe et al. examined American five-year-olds with normal body mass and overweight and found low levels of physical activity in both groups [40]. Haerens et al. [41] indicated the contribution of genetic factors in the level of physical activity of children as well as the cooperation of genetic stigma together with the impact of the environment on greater or lesser physical activity. In this regard, in this study, a focus was placed on other factors that may affect children’s BMI.

The researchers are paying more and more attention to the problem of shortened (<9 h) sleep periods in children. This problem is associated with a decrease in leptin levels and an increase in ghrelin, which can have a significant impact on appetite regulation [42]. The shorter sleep time is also associated with the high blood cortisol, which increases appetite and the accumulation of visceral fat [43], as well as a sedentary lifestyle, snacking, or watching television [44]. People who are prone to such behaviors have more time to eat caloric meals, experience fatigue during the day, and to prevent this, consume drinks and foods with higher calories [45]. The study showed that children who slept less or had a worse quality of sleep had higher BMI values. In the group of boys, the sleep variable influenced the BMI level. The boys who slept less or whose sleep quality was worse had higher BMI values. Furthermore, in children aged 11 to 15, BMI was related to sleep. The lower the sleep quality or shorter sleep, the higher the BMI value. These results may indicate the importance of sleep in the prevention and treatment of obesity in children. A meta-analysis by Ruan et al. [46] showed that among the seven studies analyzed, the group of children who slept the least (about 10 h) had a 76% higher risk of developing overweight and obesity than children sleeping longer (about 12 h).

The causes of overweight and obesity among children and adolescents can also include the influence of mass media, watching movies, or playing games on the computer, TV, or smartphone. Many children spend about 4–5 h a day performing this activity [47]. As early as preschool age, they spend more time in front of a computer or TV screen than playing outside [48]. Dietz and Gortmaker [20] were the first to observe a relationship between television viewing time and the prevalence of childhood obesity. In their subsequent studies, it was demonstrated that around 29% of people with obesity could be prevented from developing the disease by shortening their TV viewing time [49]. The results gathered showed that technological devices and Internet usage influence BMI values. In general, the children who played computer games longer had a higher body mass index. In the group of girls, the BMI value depended solely on media use. The longer the girls played computer games, or the longer they watched movies online, or used a smartphone, the higher the BMI value was. In the age group of 6 to 10, the BMI was influenced by watching movies online and using a smartphone. The longer 6-10-year-olds used the media in this way, the higher their BMI was. Such a relationship has not been observed in older children. This could indicate a particular impact of technological devices and Internet usage on this age group.

Epstein et al. [50] observed a correlation between a decrease in the incidence of overweight and obesity among children with obesity and a decrease in a sedentary lifestyle, in particular, the time spent watching TV, playing computer games. The time spent in front of the TV screen correlated positively with a larger number of meals consumed as well as the time of watching programs by parents. Another study looked at the positive relationship between the time spent in front of the screen, the number of sweet drinks consumed, and the risk of developing obesity. Less frequent TV watching and drinking fewer sweet drinks during the day were characteristic of people with lower BMI [51]. According to Caroli et al. [52], frequent TV viewing accompanied by the consumption of high-calorie snacks, reduced physical activity, and short sleeping time has an impact on the incidence of overweight and obesity in children. The regression results obtained in this study showed that it was sleep and using a smartphone that had the greatest impact on BMI in children.

This study contributed to the analysis of the effect of dietary habits, watching TV, and the amount of sleep on the BMI. One of the strengths of the study was a relatively large study group. Interestingly, the study took into account not only the time spent in front of the TV and computer but also other portable technical devices, such as tablets and smartphones. As suggested, whenever the children leave the house, they are likely to use them. Additionally, the weight and the height in this study were directly measured by a team of healthcare professionals who have extensive experience in collecting health information. This could mitigate the potential bias of recall information. There is also a number of potential limitations of the study that need to be taken into account when interpreting the results. This study was limited in terms of geographic scope and should be replicated among a larger sample across the regions. The response bias, such as social desirability, is common in self-reported questionnaires and might lead to underestimation or overestimation of the present associations. Another study limitation is that parental control over eating behavior was not assessed. It may influence the likelihood of developing addictive-like eating behaviors and food consumption by the children. The food frequency questionnaire used in our study does not check the exact amount of food eaten by the children, and the amount of certain foods eaten is the main factor responsible for overweight and obesity. In the next studies, the use of a 24-h recall questionnaire is planned. However, the most extensive cohort studies have used the food frequency questionnaire to assess dietary intake. Lastly, this study had a cross-sectional study character. Consequently, the cause and effect cannot be determined.

## 5. Conclusions

Considering various conditions discussed that may influence the BMI of early-school age children, the importance of healthy lifestyle education should be emphasized among children and parents alike. The study shows that one of the factors that may affect the BMI is the amount of sleep, playing computer games, watching movies, and using smartphones. Such education should start as early as possible and continue throughout the child’s school period. A good solution might be the simultaneous promotion of proper eating habits among parents and children, the psychological and emotional aspects included, and the support of the organizations and institutions concerned with promoting health in schools.

## Figures and Tables

**Table 1:** Characteristics of the study group.

Age	6–10 years (*N* = 194)					11–15 years (*N* = 182)					Z	*p*
Total (*N* = 376)	$\bar{x}$	SD	Me	Min.	Max.	$\bar{x}$	SD	Me	Min.	Max.		
Height (cm)	138.6	9.3	138.5	116.0	164.0	158.7	9.9	158.0	130.0	183.0	−14.3	0.000
Body mass (kg)	34.6	9.8	32.6	19.0	81.2	50.7	13.7	48.3	28.0	107.3	−11.6	0.000
BMI (kg/m2)	17.7	3.3	17.1	12.1	30.2	19.9	4.0	19.1	13.4	39.9	−5.6	0.000
BMI Centiles	53.7	31.4	55.0	0.1	99.0	54.3	30.5	54.0	0.1	99.9	−0.1	0.862
Fat tissue (%)	20.8	7.4	19.6	8.2	43.9	20.0	8.3	19.7	5.2	49.0	−1.1	0.239
Fat free mass (kg)	26.8	5.5	26.3	17.3	45.6	39.8	8.4	38.5	24.8	66.5	−13.8	0.000
Total body water (kg)	19.6	4.0	19.3	12.7	33.4	29.1	6.2	28.2	18.2	48.7	−13.8	0.000
**6–10 years (*N* = 194)**	**Boys (*N* = 99)**					**Girls (*N* = 95)**					Z	*p*
Height (cm)	138.4	9.0	138.0	116.0	164.0	138.8	9.6	139.0	121.0	162.0	−0.1	0.924
Body mass (kg)	34.8	10.0	33.0	20.6	81.2	34.4	9.6	32.0	19.0	60.5	−0.1	0.923
BMI (kg/m2)	17.8	3.3	17.0	13.3	30.2	17.6	3.4	17.2	12.1	26.5	−0.5	0.619
BMI Centiles	53.8	30.8	53.0	1.0	99.0	53.7	32.3	58.0	0.1	98.0	−0.0	0.980
Fat tissue (%)	19.6	6.9	18.5	8.7	43.9	22.0	7.6	21.4	8.2	37.7	−2.2	0.024
Fat free mass (kg)	27.4	5.6	26.7	17.8	45.6	26.2	5.3	25.7	17.3	38.9	−1.3	0.167
Total body water (kg)	20.0	4.1	19.5	13.0	33.4	19.2	3.9	18.8	12.7	28.5	−1.3	0.170
**11–15 years (*N* = 182)**	**Boys (*N* = 88)**					**Girls (*N* = 94)**					Z	*p*
Height (cm)	159.2	11.4	158.5	130.0	183.0	158.2	8.2	158.0	135.0	176.0	−0.6	0.490
Body mass (kg)	50.8	14.0	47.6	29.6	86.7	50.6	13.6	49.1	28.0	107.3	−0.1	0.899
BMI (kg/m2)	19.7	3.5	18.9	13.9	28.4	20.0	4.3	19.1	13.4	39.9	−0.4	0.692
BMI Centiles	53.8	30.1	54.0	1.0	98.0	54.7	31.1	54.5	0.1	99.9	−0.2	0.792
Fat tissue (%)	15.9	6.7	14.6	5.2	33.2	23.8	7.9	23.4	9.5	49.0	−6.4	0.000
Fat free mass (kg)	42.1	9.8	41.3	25.3	66.5	37.6	6.2	37.1	24.8	54.7	−2.8	0.004
Total body water (kg)	30.8	7.2	30.2	18.5	48.7	27.5	4.5	27.1	18.2	40.0	−2.8	0.004

$\bar{x}$—arithmetic mean; Me—median; SD—standard deviation; Z—Mann–Whitney U test result; *p*—*p*-value, indicate significant values (*p* < 0.05).

**Table 2:** Body mass categories based on body mass index (BMI).

Title		6–10				11–15				6–10		11–15		Total	
		Boys		Girls		Boys		Girls							
		*N*	%	*N*	%	*N*	%	*N*	%	*N*	%	*N*	%	*N*	%
Body weight category (WHO)	Underweight	2	2.0%	6	6.3%	2	2.3%	3	3.2%	8	4.1%	5	2.7%	13	3.5%
	Normal body mass	75	75.8%	68	71.6%	67	76.1%	69	73.4%	143	73.7%	136	74.7%	279	74.2%
	Overweight	18	18.2%	18	18.9%	18	20.5%	18	19.1%	36	18.6%	36	19.8%	72	19.1%
	Obesity	4	4.0%	3	3.2%	1	1.1%	4	4.3%	7	3.6%	5	2.7%	12	3.2%
		X^2^ = 2.404; *p*=0.493				X^2^ = 1.834; *p* = 0.608				X^2^ = 0.819; *p* = 0.845					

*n*—number of observations; %—percent; χ²—Pearson’s chi-squared test result; *p*—*p*-value, indicate significant values (*p* < 0.05).

**Table 3:** Relationship between body mass category (BMI) and selected variables—children age from 6 to 10.

Selected Variables	Age											
	6–10										Z	*p*
	Body Mass Category (BMI) According (WHO)											
	Normal Body Mass					Overweight/Obese						
	$\bar{x}$	SD	Me	Min.	Max.	$\bar{x}$	SD	Me	Min.	Max.		
Systolic pressure (mmHg)	106.12	12.88	105	67	155	110.47	16.20	111	62	141	−2.02	0.043
Diastolic pressure (mmHg)	70.83	11.90	70	48	132	72.51	8.49	72	47	87	-2.20	0.028
Time of watching movies or programs in the Internet (on an iPad, tablet, computer, smartphone) or TV per day in the weekdays (h)	1.97	0.96	2	0	5	2.05	0.95	2	0	5	−0.65	0.516
Time of watching movies or programs in the Internet (on an iPad, tablet, computer, smartphone) or TV per day on the weekend (h)	2.57	0.97	3	0	5	2.81	1.01	3	0	5	−1.10	0.270
Time of playing games (on your computer/game console /smartphone/iPad, etc.) per day in the weekdays (h)	1.18	1.09	1	0	5	1.40	1.07	1	0	5	−1.29	0.195
Time of playing games (on your computer/game console /smartphone/iPad, etc.) per day on the weekend (h)	1.90	1.10	2	0	5	2.02	1.12	2	0	5	−0.72	0.474
Using a smartphone on a typical day (*n*)	2.21	1.34	2	1	7	2.72	1.58	2	1	7	−1.99	0.047
Days with 60 min or longer physical activity (*n*)	3.67	1.83	4	0	7	3.53	2.16	3	0	7	−0.44	0.660
Time of sleeping during a 24 h period on school days (h)	9.35	0.88	9.5	6.5	11.5	9.14	0.93	9.0	6.0	12.0	−1.75	0.081
Time of sleeping during a 24 h period on weekends (h)	10.42	1.14	10.5	7.0	13.0	10.43	1.35	10.5	6.0	13.0	−0.64	0.525
Sleep quality (*n*)	1.53	0.61	1	1	4	1.58	0.54	2	1	3	−0.76	0.449

$\bar{x}$—arithmetic mean; Me—median; SD—standard deviation; Z—Mann–Whitney U test result; *p*—*p*-value, indicate significant values (*p* < 0.05); *n*—number in accordance with the possibility of answering.

**Table 4:** Relationship between body mass category (BMI) and selected variables—children age from 11 to 15.

Selected Variables	Age											
	11–15										Z	*p*
	Body Mass Category (BMI)—According WHO											
	Normal Body Mass					Overweight/Obese						
	$\bar{x}$	SD	Me	Min.	Max.	$\bar{x}$	SD	Me	Min	Max		
Systolic pressure (mmHg)	120.38	14.93	119	92	190	130.05	15.23	128	103	165	−3.60	0.000
Diastolic pressure (mmHg)	75.38	8.74	75	59	111	77.68	9.78	77	59	98	−1.59	0.111
Time of watching movies or programs in the Internet (on an iPad, tablet, computer, smartphone) or TV per day in the weekdays (h)	2.29	0.97	2	0	5	2.32	1.27	2	0	5	−0.23	0.816
Time of watching movies or programs in the Internet (on an iPad, tablet, computer, smartphone) or TV per day on the weekend (h)	3.05	1.02	3	0	5	3.07	1.19	3	0	5	−0.13	0.899
Time of playing games (on your computer / game console / smartphone / iPad, etc.) per day in the weekdays (h)	1.44	1.13	1	0	4	1.29	1.10	1	0	5	−0.82	0.410
Time of playing games (on your computer / game console / smartphone / iPad, etc.) per day on the weekend (h)	2.07	1.42	2	0	5	1.78	1.26	2	0	5	−1.22	0.222
Using a smartphone on a typical day (n)	4.01	1.42	4	1	7	4.20	1.55	4	2	7	−0.49	0.625
Days with 60 min or longer physical activity (n)	4.15	1.82	4	0	7	4.32	1.88	5	0	7	−0.68	0.497
Time of sleeping during a 24 h period on school days (h)	8.20	1.38	8.0	4.0	13.0	8.29	1.54	8.5	3.5	11.5	−0.93	0.354
Time of sleeping during a 24 h period on weekends (h)	10.02	1.47	10.0	5.0	14.0	9.76	1.98	10.0	5.5	15.5	−1.02	0.307
Sleep quality (n)	1.88	0.72	2	1	4	1.85	0.79	2	1	4	−0.52	0.602

$\bar{x}$—arithmetic mean; Me—median; SD—standard deviation; Z – Mann–Whitney U test result; *p*—*p*-value, indicate significant values (*p* < 0.05); *n*—number in accordance with the possibility of answering.

**Table 5:** The result of the general regression model for the selected parameters (independent variables were selected by forward stepwise regression procedure).

BMI		Non-Standardized Coefficients		Standardized Coefficients	t	*p*	F	*p*
		***B***	SE	β				
Total	(Constant)	2.91	0.01		302.47	0.000	12.88	0.000
	Sleep	−0.03	0.01	−0.18	−3.36	0.001		
	Media—playing games	0.02	0.01	0.13	2.42	0.016		
Girls	(Constant)	2.90	0.02		192.22	0.000	9.18	0.000
	Media—playing games	0.05	0.01	0.25	3.62	0.000		
	Media—watching movies and using a smartphone	0.03	0.02	0.15	2.12	0.036		
Boys	(Constant)	2.92	0.01		224.26	0.000	8.31	0.004
	Sleep	−0.04	0.01	−0.21	−2.88	0.004		
6–10 years	(Constant)	2.87	0.01		216.45	0.000	4.57	0.034
	Media—watching movies and using a smartphone	0.03	0.01	0.15	2.14	0.034		
11-15 years	(Constant)	2.96	0.01		202.36	0.000	5.11	0.025
	Sleep	−0.03	0.01	−0.17	−2.26	0.025		

SE—standard error; *B*—regression coefficient; *p*—significance of regression coefficient; β—standardized regression coefficient; t—Student *t*-test; *—indicate significant values (*p* < 0.05); F—Fisher’s test result.

**Table 6:** Relationship between body mass category (BMI) and frequency of food intake.

Selected Food Items	Body Mass Category (BMI) According WHO										Z	*p*
	Normal Body Mass					Overweight/Obese						
	$\bar{x}$	SD	Me	Min.	Max.	$\bar{x}$	SD	Me	Min.	Max.		
Legumes (e.g., beans, lentils, chickpeas)	1.61	1.17	1	1	7	1.76	1.24	1	1	7	−1.39	0.164
Potatoes (cooked)	2.81	1.23	3	1	7	2.87	1.45	2	1	7	−0.33	0.741
Raw vegetables (mixed salad, carrot, fennel, cucumber, lettuce, tomato)	2.80	1.51	3	1	7	3.19	1.72	3	1	7	−1.82	0.068
Fresh fruits (also as freshly squeezed juice)	3.54	1.77	3	1	7	3.52	1.78	3	1	7	−0.17	0.862
Water (tap water, carbonated water, plain water)	4.89	1.97	6	1	7	5.01	1.98	6	1	7	−0.74	0.461
Fruit juices (100% fruit), packaged (orange juice, apple juice)	3.25	1.70	3	1	7	3.37	1.91	3	1	7	−0.11	0.916
Carbonated sugar sweetened drinks (e.g., coca cola, Fanta)	1.85	1.27	1	1	7	1.64	1.20	1	1	7	−1.86	0.063
Diet carbonated drinks, (e.g., diet cola)	1.46	1.06	1	1	7	1.40	1.12	1	1	7	−1.03	0.302
Tea, herbal tea, and similar—unsweetened	2.45	1.66	2	1	7	2.46	1.63	2	1	7	−0.22	0.826
Tea, herbal tea, and similar—sweetened (e.g., addition of sugar, honey, etc.)	2.78	1.67	2	1	7	2.76	1.77	2	1	7	−0.37	0.713
Plain unsweetened milk	2.32	1.51	2	1	7	2.45	1.60	2	1	7	−0.76	0.448
Plain unsweetened yoghurt or kefir	1.75	1.17	1	1	7	1.56	0.88	1	1	6	−0.94	0.349
Sweet and flavored yoghurt and fermented milk beverages (e.g., Actimel^®^, LC1^®^)	2.19	1.28	2	1	7	2.04	1.00	2	1	6	−0.40	0.686
Boiled or poached eggs	2.03	1.35	2	1	7	1.94	0.95	2	1	6	−0.61	0.544
Sliced cheese	2.24	1.28	2	1	7	2.10	1.08	2	1	7	−0.51	0.610
Spreadable cheese	1.97	1.12	2	1	7	1.80	0.95	2	1	7	−0.99	0.320
Fish, boiled, grilled, oven baked, raw	1.56	0.94	1	1	7	1.42	0.84	1	1	7	−1.40	0.162
Fish, fried and/or coated	1.60	0.93	1	1	7	1.43	0.66	1	1	4	−1.47	0.141
Cold cuts and preserved, ready to cook meat product	2.92	1.51	3	1	7	3.06	1.55	3	1	7	−0.88	0.379
Meat, boiled, grilled, oven baked, without coating, not fried	2.32	1.27	2	1	7	2.13	1.19	2	1	7	−1.38	0.168
Fried meat (beef, pork)	2.35	1.38	2	1	7	2.30	1.14	2	1	7	−0.47	0.637
Poultry, boiled, grilled, oven baked, without coating not fried	2.00	1.22	2	1	7	1.93	1.03	2	1	7	0.00	0.998
Fried poultry	2.04	1.27	2	1	7	1.82	0.79	2	1	4	−0.51	0.609
Butter, margarine on bread	3.42	1.71	3	1	7	3.27	1.74	3	1	7	−0.80	0.424
Nuts and seeds	2.05	1.26	2	1	7	1.86	1.22	2	1	7	−1.62	0.106
White bread, white roll, white crispbread	3.27	1.64	3	1	7	3.32	1.76	3	1	7	−0.12	0.904
Whole meal bread, dark roll, dark crispbread	2.39	1.39	2	1	7	2.57	1.42	2	1	7	−1.23	0.218
Pasta, noodles, rice and other cereals, white, refined	2.22	1.19	2	1	7	2.07	1.32	2	1	7	−1.82	0.069
Whole meal pasta, noodles, brown rice and other cereals, unrefined	1.69	1.10	1	1	7	1.54	0.97	1	1	7	−1.17	0.243
Sweetened or sugar added breakfast cereals and sweetened crisp muesli	2.24	1.29	2	1	7	2.12	1.40	2	1	7	−1.33	0.183
Not homemade hamburger, hot dog, kebab, wrap, falafel, sandwiches	1.83	1.31	1	1	7	1.64	1.26	1	1	7	−1.53	0.127
Snacks like crisps, corn crisps, popcorn, etc.	1.99	1.31	2	1	7	1.80	1.23	1	1	7	−1.48	0.140
Snacks like candies, loose candies, marshmallow	2.00	1.28	2	1	7	1.76	1.16	1	1	7	−1.80	0.071
Snacks like biscuits, packaged cakes, or pastries and puddings	2.13	1.26	2	1	7	1.96	1.07	2	1	7	−0.88	0.377

$\bar{x}$—arithmetic mean; Me—median; SD—standard deviation; Z—Mann–Whitney U test result; *p*—*p*-value, indicate significant values (*p* < 0.05).

## Supplementary Materials

The following are available online at https://www.mdpi.com/1660-4601/17/20/7492/s1.
