# Supplementary material for: Children’s Body Mass Index Depending on Dietary Patterns, the Use of Technological Devices, the Internet and Sleep on BMI in Children
Source: Int J Environ Res Public Health. 2020 Oct 15;17(20):7492. doi: 10.3390/ijerph17207492 (PMC7650693; doi:10.3390/ijerph17207492)
Supplement: Supplementary file 1 [file ijerph-17-07492-s001.zip › Questionnaire template.docx]

**ID Participant**

# Socio-demographic characteristics

1. What is your sex?

Male **⚫**_1_

Female _2_

1. What is Your date of birth?

………. Day ……. Month ……….Year

**I PART**

**FOOD FREQUENCY QUESTIONNAIRE**

1. **In the last month, how many times did your child eat or drink the following food items? Please indicate only foods and drinks you know about, i.e. what your child ate in your presence.**

*Please tick one answer per line.*

| **In the last month…** | | Never/ less  than once a week | 1-3 times  a  week | 4-6  times  a  week | 1  time per  day | 2  times  per  day | 3  times  per  day | | 4 or  more  times  per  day | | |
| --- | --- | --- | --- | --- | --- | --- | --- | --- | --- | --- | --- |
| **Vegetables** | | | | | | | | | | | |
| Legumes (e.g. beans, lentils,  Chickpeas) | | **⚫** _1_ | **⚫** _2_ | **⚫** _3_ | **⚫** _4_ | **⚫** _5_ | **⚫** _6_ | | **⚫** _7_ | | |
| Potatoes (cooked) | | **⚫** _1_ | **⚫** _2_ | **⚫** _3_ | **⚫** _4_ | **⚫** _5_ | **⚫** _6_ | | **⚫** _7_ | | |
| Raw vegetables (mixed  salad, carrot, fennel,  cucumber, lettuce,  tomato) | | **⚫** _1_ | **⚫** _2_ | **⚫** _3_ | **⚫** _4_ | **⚫** _5_ | **⚫** _6_ | | **⚫** _7_ | | |
| **Fresh fruits** | |  |  |  |  |  |  | |  | | |
| Fresh fruits (also as freshly  squeezed juice) | | **⚫** _1_ | **⚫** _2_ | **⚫** _3_ | **⚫** _4_ | **⚫** _5_ | **⚫** _6_ | | **⚫** _7_ | | |
| **Drinks** | |  |  |  |  |  |  | |  | | |
| Water (tap water,  carbonated water, plain  water) | | **⚫** _1_ | **⚫** _2_ | **⚫** _3_ | **⚫** _4_ | **⚫** _5_ | **⚫** _6_ | | **⚫** _7_ | | |
| Fruit juices (100% fruit),  packaged (orange juice,  apple juice) | | **⚫** _1_ | **⚫** _2_ | **⚫** _3_ | **⚫** _4_ | **⚫** _5_ | **⚫** _6_ | | **⚫** _7_ | | |
| Carbonated sugar sweetened  drinks (e.g. coca cola, fanta) | | **⚫** _1_ | **⚫** _2_ | **⚫** _3_ | **⚫** _4_ | **⚫** _5_ | **⚫** _6_ | | **⚫** _7_ | | |
| Diet carbonated drinks, (e.g.  diet cola) | | **⚫** _1_ | **⚫** _2_ | **⚫** _3_ | **⚫** _4_ | **⚫** _5_ | **⚫** _6_ | | **⚫** _7_ | | |
| Tea, herbal tea and similar:  a) unsweetened  b) sweetened (e.g. addition of sugar, honey etc.) | | **⚫**  _1_  **⚫**  _1_ | **⚫**  _2_  **⚫**  _2_ | **⚫**  _3_  **⚫**  _3_ | **⚫**  _4_  **⚫**  _4_ | **⚫**  _5_  **⚫**  _5_ | **⚫**  _6_  **⚫**  _6_ | | | **⚫**  _7_  **⚫**  _7_ |  |
| **Breakfast cereals** | |  |  |  |  |  |  | | |  |  |
| Sweetened or sugar added  breakfast cereals and  sweetened crisp muesli | | **⚫** _1_ | **⚫** _2_ | **⚫** _3_ | **⚫** _4_ | **⚫** _5_ | **⚫** _6_ | | | **⚫** _7_ |  |
| **Milk** | |  |  |  |  |  |  | | |  |  |
| Plain unsweetened milk | | **⚫**  _1_ | **⚫**  _2_ | **⚫**  _3_ | **⚫**  _4_ | **⚫**  _5_ | **⚫**  _6_ | | | **⚫**  _7_ |  |
| **Yoghurt** | |  |  |  |  |  |  | | |  |  |
| Plain unsweetened yoghurt  or kefir | | **⚫**  _1_ | **⚫**  _2_ | **⚫**  _3_ | **⚫**  _4_ | **⚫**  _5_ | **⚫**  _6_ | | | **⚫**  _7_ |  |
| Sweet and flavouredyoghurt  and fermented milk  beverages (e.g. Actimel®,  LC1®) | | **⚫**  _1_ | **⚫**  _2_ | **⚫**  _3_ | **⚫**  _4_ | **⚫**  _5_ | **⚫**  _6_ | | | **⚫**  _7_ |  |
| **Fish** | | | | | | | | | | |  |
| Fish, boiled, grilled, oven baked, raw | | **⚫**  _1_ | **⚫**  _2_ | **⚫**  _3_ | **⚫**  _4_ | **⚫**  _5_ | **⚫**  _6_ | | | **⚫**  _7_ |  |
| Fish, fried and/or coated | | **⚫**  _1_ | **⚫**  _2_ | **⚫**  _3_ | **⚫**  _4_ | **⚫**  _5_ | **⚫**  _6_ | | | **⚫**  _7_ |  |
| **Meat and meat products** | | | | | | | | | | |  |
| Cold cuts and preserved,  ready to cook meat product | | **⚫**  _1_ | **⚫**  _2_ | **⚫**  _3_ | **⚫**  _4_ | **⚫**  _5_ | **⚫**  _6_ | | | **⚫**  _7_ |  |
| Meat, boiled, grilled, oven baked, without coating, not fried (beef, pork) | | **⚫**  _1_ | **⚫**  _2_ | **⚫**  _3_ | **⚫**  _4_ | **⚫**  _5_ | **⚫**  _6_ | | | **⚫**  _7_ |  |
| Fried meat (beef, pork) | | **⚫**  _1_ | **⚫**  _2_ | **⚫**  _3_ | **⚫**  _4_ | **⚫**  _5_ | **⚫**  _6_ | | | **⚫**  _7_ |  |
| Poultry, boiled, grilled, oven baked, without coating ,not fried | | **⚫**  _1_ | **⚫**  _2_ | **⚫**  _3_ | **⚫**  _4_ | **⚫**  _5_ | **⚫**  _6_ | | | **⚫**  _7_ |  |
| Fried poultry | | **⚫**  _1_ | **⚫**  _2_ | **⚫**  _3_ | **⚫**  _4_ | **⚫**  _5_ | **⚫**  _6_ | | | **⚫**  _7_ |  |
| **Eggs and mayonnaise** | | | | | | | | | | |  |
| Boiled or poached eggs | **⚫**  _1_ | | **⚫**  _2_ | **⚫**  _3_ | **⚫**  _4_ | **⚫**  _5_ | **⚫**  _6_ | **⚫**  _7_ | | |  |
| **Cheese** | | | | | | | | | | |  |
| Sliced cheese | **⚫**  _1_ | | **⚫**  _2_ | **⚫**  _3_ | **⚫**  _4_ | **⚫**  _5_ | **⚫**  _6_ | **⚫**  _7_ | | |  |
| Spreadable cheese | **⚫**  _1_ | | **⚫**  _2_ | **⚫**  _3_ | **⚫**  _4_ | **⚫**  _5_ | **⚫**  _6_ | **⚫**  _7_ | | |  |
| **Spreadable products** | | | | | | | | | | |  |
| Butter, margarine on bread | **⚫**  _1_ | | **⚫**  _2_ | **⚫**  _3_ | **⚫**  _4_ | **⚫**  _5_ | **⚫**  _6_ | **⚫**  _7_ | | |  |
| **Cereal products** | | | | | | | | | | |  |
| White bread, white roll,  white crispbread | **⚫**  _1_ | | **⚫**  _2_ | **⚫**  _3_ | **⚫**  _4_ | **⚫**  _5_ | **⚫**  _6_ | **⚫**  _7_ | | |  |
| Whole meal bread, dark roll, dark crispbread | **⚫**  _1_ | | **⚫**  _2_ | **⚫**  _3_ | **⚫**  _4_ | **⚫**  _5_ | **⚫**  _6_ | **⚫**  _7_ | | |  |
| Pasta, noodles, rice and  other cereals, white, refined | **⚫**  _1_ | | **⚫**  _2_ | **⚫**  _3_ | **⚫**  _4_ | **⚫**  _5_ | **⚫**  _6_ | **⚫**  _7_ | | |  |
| Whole meal pasta, noodles,  brown rice and other  cereals, unrefined | **⚫**  _1_ | | **⚫**  _2_ | **⚫**  _3_ | **⚫**  _4_ | **⚫**  _5_ | **⚫**  _6_ | **⚫**  _7_ | | |  |
| Not homemade hamburger,  hot dog, kebab, wrap,  falafel, sandwiches | **⚫**  _1_ | | **⚫**  _2_ | **⚫**  _3_ | **⚫**  _4_ | **⚫**  _5_ | **⚫**  _6_ | **⚫**  _7_ | | |  |
| **Snacks** | | | | | | | | | | |  |
| Nuts and seeds | **⚫**  _1_ | | **⚫**  _2_ | **⚫**  _3_ | **⚫**  _4_ | **⚫**  _5_ | **⚫**  _6_ | **⚫**  _7_ | | |  |
| Snacks like crisps, corn  crisps, popcorn etc. | **⚫**  _1_ | | **⚫**  _2_ | **⚫**  _3_ | **⚫**  _4_ | **⚫**  _5_ | **⚫**  _6_ | **⚫**  _7_ | | |  |
| Snacks like candies, loose  candies, marshmallow | **⚫**  _1_ | | **⚫**  _2_ | **⚫**  _3_ | **⚫**  _4_ | **⚫**  _5_ | **⚫**  _6_ | **⚫**  _7_ | | |  |
| Snacks like biscuits,  packaged cakes, or pastries  and puddings | **⚫**  _1_ | | **⚫**  _2_ | **⚫**  _3_ | **⚫**  _4_ | **⚫**  _5_ | **⚫**  _6_ | **⚫**  _7_ | | |  |

**II PART**

**SLEEP**

1. **What is the amount of time you sleep during a 24 hour period on school days?**

…….. ………. **hours** and …………….**minutes** per night

…………………….**hour(s)** and …………………**minutes** napping during daytime

1. **What is the amount of time you sleep during a 24 hour period on weekends?**

…….. ………. **hours** and …………….**minutes** per night

…………………….**hour(s)** and …………………**minutes** napping during daytime

1. **During the past month, how would you rate your sleep quality overall?**

*Please tick one answer.*

Very good **⚫**_1_

Fairly good **⚫**_2_

Fairly bad **⚫**_3_

Very bad **⚫**_4_

**III PART**

**USE OF MEDIA**

1. **How long do you usually watch movies or programs on the Internet (on an iPad, tablet, computer, smartphone) or on the television per day?**

*Please tick one answer per line.*

Not Less than Between Between Between More than

at all 30 min. 30 min. 2 and 3 hrs. 3 and 6 hrs. 6 hrs.

per day and 2 hrs. per day per day per day

per day

Weekdays  **⚫**_0_  **⚫**_1_  **⚫**_2_   **⚫**_3_  **⚫**_4_   **⚫**_5_

Weekends  **⚫**_0_  **⚫**_1_  **⚫**_2_   **⚫**_3_  **⚫**_4_   **⚫**_5_

1. **How long do you usually play electronic games (at a computer, game console, smartphone, iPad, etc.) per day?**

*Please tick one answer per line.*

Not Less than Between Between Between More than

at all 30 min. 30 min. 2 and 3 hrs. 3 and 6 hrs. 6 hrs.

per day and 2 hrs. per day per day per day

per day

Weekdays  **⚫**_0_  **⚫**_1_  **⚫**_2_   **⚫**_3_  **⚫**_4_   **⚫**_5_

Weekends  **⚫**_0_  **⚫**_1_  **⚫**_2_   **⚫**_3_  **⚫**_4_   **⚫**_5_

**3. How often do you use your smartphone on a typical day?**

I don’t have access to a smartphone **⚫**_1_

Less than 5 times per day **⚫**_2_

6 – 10 times per day **⚫**_3_

11 – 20 times per day **⚫**_4_

21 – 50 times per day **⚫**_5_

51 – 100 times per day **⚫**_6_

More than 100 times per day **⚫**_7_

**IV PART**

**Estimation of overall PA**

1. During the last week, in how many days have you performed 60 minutes or more of physical activity that was enough to increase your breathing rate? This may include sport, exercise and brisk walking or cycling for recreational purposes or on the way to these places.

0 days 1 day 2 days 3 days 4 days 5 days 6 days 7 days

**⚫** _0_  **⚫** _1_  **⚫** _2_   **⚫** _3_  **⚫** _4_   **⚫** _5_  **⚫** _6_  **⚫** _7_
